# Protocol for a study on the mental health and well-being of “Forgotten” first responders in Canada

**DOI:** 10.1371/journal.pone.0346019

**Published:** 2026-04-03

**Authors:** Diana McGlinchey, Alyssa Ferns, Chris William Martin, Ashley Murfin, Kyle Killian, Jenna Row, Jalynn Countaway, McKenna Bellemare, Ocean Naneff, Amaya Purewal

**Affiliations:** 1 Victimology Research Centre, Algonquin College, Ottawa, Ontario, Canada; 2 Marriage and Family Therapy, Capella University, Minneapolis, Minnesota, United States of America; PLOS: Public Library of Science, UNITED KINGDOM OF GREAT BRITAIN AND NORTHERN IRELAND

## Abstract

In addition to traditional first responders such as police, firefighters, and paramedics, there are many other workers who carry an occupational responsibility to respond to trauma. Such professions include but are not limited to correctional officers, frontline homeless shelter staff and security, harm reduction and supervised consumption site workers, and search and rescue teams. Research has demonstrated that individuals in these professions experience similar trauma-responses to first responders, albeit often without the same support and safeguards. The study described in this protocol paper aims to explore the burnout, compassion fatigue, and resilience experienced by these overlooked, “forgotten” first responders. Using a mixed methods approach, including an online survey, focus groups, and semi-structured interviews, the study will investigate burnout rates, compassion fatigue, coping strategies, vicarious resilience, and resources and barriers to well-being for the people doing this important work.

## Introduction

In recent years, there has been a significant increase in research focusing on the impact of vicarious trauma and burnout in frontline helping professions in Canada (for example, the mental health of police officers [1]; firefighters [2]; and victim service providers [3]). However, there are several occupations whose members are exposed to the same level of trauma, but are overlooked in mental health research (i.e., provincial and federal correctional officers, frontline homeless shelter staff and security, harm reduction and supervised consumption site workers, search and rescue teams, 911/fire/enforcement dispatchers, crime scene cleanup staff, tow-truck drivers, volunteer firefighters, lifeguards, and cultural heritage first responders). Given our understanding of the importance of protecting workers’ well-being to promote and maintain a resilient workforce, it is imperative to have a thorough and robust understanding of the impact of this type of work across multiple forgotten first responder positions. The anticipated outcomes of this project will determine current rates of burnout for individuals serving in trauma response roles in Canada; create and provide training for organizations and individuals to prevent burnout and promote resilience; and create and deliver accessible knowledge dissemination. Through these outcomes, we expect to benefit our community partners and greater community through an understanding of the impact of trauma work on non-traditional first responders. These outcomes will be supported by detailed data informed by those who have lived experiences. Algonquin College, the VRC, the PPSI, and the School of Health Studies will find benefits through the ability to build relationships with community partners outside of the typical victim/survivor support organizations. The PPSI and School of Health Studies can implement future findings into their programs as they directly educate students in moving to work in trauma response fields. Finally, this study will further the field of victimology (which seeks to understand the experiences of individuals and communities who are affected by trauma) by expanding what we know about the impact of trauma on non-primary victims.

### New knowledge development

This project will develop new knowledge on the impact of trauma response work in populations who are not typically included in first responder mental health research, with a specific focus on the Canadian population. Specifically, we will identify overall wellness and barriers that people who work in trauma response face in accessing mental health support. Our findings can be used to develop tools, training, and other resources that foster positive mental health outcomes for people who work in trauma response fields. This knowledge will be incorporated into college curricula, training modules, and written reports and articles.

## Literature review

### Definitions of forgotten first responders

The job category “first responder” typically includes police, firefighters, and paramedics; for the proposed legal definition in Canada, see Bill C-345 [4], which seeks to amend the *Criminal Code of Canada* (which currently refers to peace officers) to include “emergency medical technician, paramedic and firefighter”. Correspondingly, research related to the mental health of first responders is typically limited to these roles. This body of research looks at the impact of responding to trauma (such as motor vehicle accidents, violent crime, suicides, fires, responding in the COVID-19 pandemic, etc.) (see, for example, [1,2,5–7]). In fact, in response to the high rates of mental health issues among public safety personnel, the Canadian government developed “*Supporting Canada’s Public Safety Personnel: An Action Plan on Post-Traumatic Stress Injuries*” [8]. Its goal was to improve risk mitigation, diagnosis, and treatment and supported a new national research consortium between the Canadian Institutes for Health Research (CIHR) and the Canadian Institute for Public Safety Research and Treatment (CIPSRT) [2]. It is evident that there has been a shift from policymakers to take trauma work and its impact seriously for the traditional first responders. However, many other professions experience occupational exposure to the same traumas (correctional staff, frontline staff at homeless shelters, search and rescue teams, disaster cleanup crews, tow truck drivers, lifeguards, cultural heritage first responders, and volunteer firefighters), but are rarely included in Canadian research literature, leading to the term “forgotten first responders”.

### Comparisons of work to other first responders

Forgotten first responders are directly exposed to traumatic scenes through the work that they do. For example, 911/fire/law enforcement dispatchers are often the first point of contact for someone in crisis [9]. Correctional staff are first on scene for physical altercations, self-injuries, suicides, homicides, and overdoses within correctional institutions [10]. Similarly, frontline staff at homeless shelters respond to similar incidents in their shelters [11], and workers at harm reduction and supervised consumption sites respond to overdoses and other medical emergencies (for example, [12]). Workers within shelters or those at supervised consumption sites report a range of stressors including financial insecurity, a lack of respect, lack of significant wages and benefits, and constant exposure to death and trauma [13, 14]. Search and rescue teams may find missing persons who died by suicide, experienced animal attacks, or were victims of homicide, natural or manmade disaster, or misadventure [15]. Likewise, lifeguards are often first on scene for accidents, natural and manmade disasters, and misadventures on the water [16]. Disaster cleanup crews respond to the aftermath of a number of tragic circumstances (such as homicide, suicide, and overdose deaths) and while the victim(s) may not be on scene, the exposure to the scene of the incident could be enough to cause vicarious trauma [17]. Similarly, tow truck drivers clean up the aftermath of car crashes, which may expose them to graphic visuals [18]. Uniquely, cultural heritage first responders are tasked with salvaging cultural and historical artifacts after a disaster event, and may face additional pressure to preserve items of significance while being exposed to destruction and others’ suffering [19].

### The nature of 24/7 work

Like traditional first responders, forgotten first responders work in organizations that operate on a 24/7/365 schedule. The implications of this type of schedule are well documented: through a literature review, Brown et al. (2020) [20] found that shift work correlated to a reduction in sleep quality, depressed mood and anxiety, substance use, impairments in cognition, lower quality of life, and an increase in suicidal ideation.

### Impact of trauma work

Working in an occupation that exposes a responder to trauma will have an effect on people who do this work. Decreases in positive mental health outcomes, posttraumatic stress disorder (PTSD), secondary traumatic stress, burnout, and compassion fatigue are commonly cited consequences of helping professions [21, 22]. It is important to note that not all people who are exposed to trauma experience negative mental health outcomes [23]. However, people who are exposed to trauma through their work have higher rates of adverse mental health outcomes than do those working in other professions [24]. For example, one study found that the prevalence rate for people exposed to trauma through their work in Canada is 44.5% [25] compared to an approximate 8% lifetime prevalence of PTSD in the general population [26]. The Canadian Mental Health Association notes that traditional first responders experience PTSD at two times the rate versus the general population [27].

There are significant economic factors associated with PTSD and burnout. Mental illness in the Canadian labour force is estimated to result in productivity losses of between $2.75 and 8.25 billion CAD each year [28]. While it’s not possible to estimate the amount specific to forgotten first responders who are off work due work-related stress injuries, it is reasonable to conclude that the number is not zero, and any reduction in that number would be an economic benefit to Canada.

### Limited data

The data that currently exists on this topic is based on international samples (for example, [21, 29]) or on disparate sectors with small sample sizes (for example, [19]), or effects of specific disaster events (for example, [29, 30]), or a combination of these limitations. For example, a study of search and rescue volunteers in Colorado, United States found that burnout and stress levels among SAR volunteers are higher than the general population and comparable to other first responders [31], but its sample size and specific region make it difficult to generalize these findings. The gap in knowledge in a Canadian context points to the need for a national study, with an expanded definition of participants.

### Theoretical framework

This project will be informed by disaster victimization [32]. Taylor uses this framework to assign different classifications of victims based on proximity to the victimization event. In this framework, primary victims are the direct victims; secondary victims are those in the close network of primary victims (such as family members, neighbours, friends, and colleagues); and tertiary victims are people who experience trauma through their occupational responsibility to assist victims (for example, first responders, social workers, and victim service providers). The benefit to this classification system is that it allows for resources to be allocated as needed to meet the needs of each classification. In the context of this project, findings will be aligned with the needs of tertiary victims.

## Materials and methods

### Study design

This study has been designed in consultation with supporting organizations and collaborators to assess research needs within their sectors and to ensure that the project is as useful as possible. The study will include an online survey, semi-structured interviews, and focus groups. All instruments will be available in both of Canada’s official languages (English and French).

### Participant sample and data collection

We aim to reach a convenience sample of 1,000 forgotten first responders by having our research team snowball our recruitment text throughout their networks. Our research team will use social media, word of mouth, and professional contact lists.

#### Inclusion and exclusion criteria.

Inclusion criteria for this study will be based on the following three yes or no screening questions at the start of the survey: 1) Are you 18 years of age or older?; 2) Does your role require you to respond to traumatic scenes or events (including responding to phone or internet communications)?; and 3) Do you consent to participate? If a participant selects “no” for any of these questions, they will be taken to a disqualification page. Additionally, in a later section titled About Your Work Experience, participants will have the option to write in their position title. Position titles which are aligned with traditional first responder roles (fire, police, paramedic) or common healthcare roles (nurses, doctors, dentists) will be removed during data clean up.

### Survey, interview, and focus group data collection

Survey data will be collected using Survey Monkey. The survey will allow participants to indicate if they would like to be contacted to participate in a follow up interview.

We will conduct in-depth interviews, either in person, by phone, or virtual meeting platform (such as Zoom or Teams). These interviews will follow a semi-structured interview guide, which allows participants to co-create the narratives around topics they value most [33]. The possible themes that may be explored within the interviews and focus groups include current workplace support systems, worker well-being and resilience, as well as vicarious trauma. These themes will be supported by appropriate scales and literature which will provide the opportunity for open dialogue and discussion with participants.

We plan to hold focus groups with workers in different forgotten first responder fields to generate group discussions and to facilitate information sharing. We have the capacity to facilitate both in-person and virtual focus groups.

### Survey measures

#### Demographics and organizational characteristics.

Demographic questions will include age, ethnic or cultural origins, gender and sexual identity, relationship status, religious affiliations, education qualifications, and family structure. Participants will also be asked questions regarding organizational characteristics specific to their workplace or profession. These will include which province or territory it is located in and the type (e.g., government vs non-government), as well as descriptors (e.g., urban, rural, etc.) and the size of the organization (e.g., no workers, < 5 workers, and 100 + workers). We will also ask about time spent within the position, feelings of preparedness for individual caseloads, and unionization.

#### Organizational supports.

This section will ask participants about the support they receive from their organization. Sense of morale will be measured by 10 items adapted by Killian (2008) [34], five items measuring participants’ sense of autonomy or locus of control at work from Trudeau et al. (2001) [35], eleven items measuring organizational cynicism [36], and six items measured worker alienation [37]. These items utilize a 5-point Likert Scale ranging from 0 (strongly disagree) to 4 (strongly agree). Killian (2008) [34] found that the variables of job autonomy/locus of control at work, support at work, and worker morale were significant predictors of professional quality of life.

#### Job satisfaction.

This section will ask participants about their satisfaction level with their jobs by using the scale Job Satisfaction Survey (JSS) [38] and the Perceived Stress Scale (PSS) [39]. The Job Satisfaction Survey (JSS) is a 36-item survey used to gauge how employees feel about different facets of their jobs [38]. The JSS assesses nine factors: communication, coworkers, nature of work, operating procedures, fringe benefits, promotion, supervision, pay, and contingent rewards [40]. Four items are used to test each feature, and answers are recorded on a 6-point Likert scale from “Disagree very much” to “Agree very much” [38]. The JSS generates individual aspect ratings and an overall job satisfaction scale by summating pertinent components [38]. This study utilizes a 19-item version proposed by Li and Huang (2017) [40] who conducted exploratory and confirmatory factor analyses using the full 36-item JSS with a sample of child welfare workers.

The Perceived Stress Scale (PSS) is a 10-item survey using a 5-point Likert scale to assess feelings of stress within the past month [39]. Respondents will be asked to rate “how often” they felt a certain way within the last 30 days based on these 10 questions. Specifically, this measurement tool aims to measure the predictability, controllability, and possible level of overload of a participant's face in life. To aid with understanding, this scale is also free of context-specific questions, which allows for more interpretation.

#### Professional quality of life.

Professional quality of life will be measured using the Professional Quality of Life: Compassion Satisfaction and Fatigue Version 5 (ProQOL). This scale is aimed to be a reflective measure of the participant’s opinion and experiences as a “helper” in their current work situation within the past 30 days [41]. It is measured on a 5-point Likert scale ranging from 1 (Never) to 5 (Very Often). Using three discrete scales, this measurement looks at 1) compassion satisfaction, 2) compassion fatigue, and 3) burnout in relation to the participant’s field of work. Scores below 22 are considered ‘Low,’ those between 23–41 are considered ‘Average,’ and scores above 42 are considered ‘High.’ Therefore, a greater score will indicate a more satisfactory feeling within the question or section’s context. For example, a higher score within the compassion satisfaction section would indicate the respondent feels positively about their contributions to their job [41].

The ProQOL has been adapted for this study by reducing it to a briefer, 20-item version that retains high internal consistency for each subscale based on reliability analyses conducted as part of a previous research study examining vicarious resilience in Canadian victim service workers [42]. This measurement includes three subscales: 1) Brief Burnout subscale, 2) Brief Compassion Fatigue subscale, and 3) Brief Compassion Satisfaction subscale. Using a 5-point Likert scale, participants will rank their opinions on how they feel after a shift at work with topics ranging from emotional exhaustion, traumatic stress, to feelings of satisfaction and success.

#### Burnout.

This section will ask participants about their current job experience and its balance with their energy and well-being using the Workplace Stress Scale (WSS) and the Work Drain scale [43] and a 4-item measure of burnout adapted from Stamm (2010) [41]. The Workplace Stress Scale (WSS) is a 5-point Likert scale that ranges from 1 (never) to 5 (very often) based on eight current job experience-based questions on topics such as job safety, workload, emotional well-being, work-life balance, and job satisfaction. The final score is calculated by adding all replies and can range from <15–40. The final score will indicate the severity of stress per participant; a score of <15 is equivalent to one having a calm demeanour, whereas a score ranging between 31–40 is equivalent to one’s stress level being dangerous to their physical and mental health [44]. Work Drain is a three-item scale that allows participants to rate how much energy they have for activities in their personal lives at the end of their work day [43]. It uses a 5-point Likert scale that ranges from 0 (strongly disagree) to 4 (strongly agree).

#### Vicarious trauma.

Vicarious trauma will be measured with the Vicarious Trauma Scale (VTS), which is an eight-item self-report measure used to assess the subjective levels of discomfort experienced by professionals who work with traumatized people [45]. Each question is graded on a 7-point Likert scale, from 1 (Strongly Disagree) to 7 (Strongly Agree), indicating the level to which respondents experience symptoms of vicarious trauma at work [45]. This scale assesses major features of vicarious trauma, including exposure to disturbing information, the emotional effect of client interactions, trouble coping with job-related content, intrusive thoughts outside of work, feelings of powerlessness, and general emotional tiredness [45]. Higher scores indicate greater levels of distress, suggesting a higher risk of vicarious trauma [45].

#### Mental health.

Mental health will be measured with the Generalized Anxiety Disorder 7 (GAD-7) [46], the SPAN (PTSD Measure) [47], the Subtle Screening of Suicidal Ideation (SSSI), and the Patient Health Questionnaire – 5 (PHQ-5) [46, 48, 49]. The Generalized Anxiety Disorder 7 (GAD-7) is a 7-item questionnaire based on the criteria for anxiety in the DSM-5 [50]. Each item is measured by a 4-point Likert scale where participants will rate how much each item bothered them over the past two weeks, from 0 (not at all) to 3 (nearly every day). Scores are summed to find the total, with higher scores indicating high anxiety levels.

The SPAN Measure is a four-item self-report scale developed to assess PTSD [47]. Derived from the 17-item Davidson Trauma Scale (DTS), Meltzer-Brody et al. sought to create a shortened version of this scale to serve as a screening tool. Specifically, the four items derived from the original scale, which are included in the shortened version are startle, anger, numbness, and physical upset at exposure to reminders of the trauma. Participants rank each item on a scale of 0 (not at all distressing) to 4 (extremely distressing). This measure is then assessed on a “likelihood ratio”; the likelihood of the disease being present when test results are calculated and a positive test result is found. Therefore, a score of 3 is considered “moderately positive,” whereas a score of 10 is considered “strongly positive”.

The SSSI is a 19-item self-report scale developed to measure the severity of psychological pain (or psychache) and is a proxy measure for suicidal ideation [48]. Killian (2025) reported excellent reliability (α  =  .93) and validity for the SSSI. A Receiver Operator Characteristic curve identified a cut-off score of 35 with a sensitivity of  .937 and specificity of  .81, indicating the instrument successfully identifies those with and without suicidal thoughts.

This study will use the PHQ-5, which is a five-item version of the Patient Health Questionnaire [46, 49, 51]. The PHQ-5 includes 2-item measures of anxiety and depression and one item measuring suicidal ideation. Two additional items from the Interpersonal Needs Questionnaire and three items from the Columbia-Suicide Severity Rating Scale were added to create a 6-item measure of suicidal ideation.

This study will use six items of the Chalder Fatigue Scale to measure symptoms of physical drain, energy, and mental drain [52]. Items are rated on a 4-point Likert scale (0 = “better than usual”, 1 = “no more than usual”, 2 = “worse than usual”, 3 = “much worse than usual”) with higher scores indicating greater fatigue.

Finally, the Moral Injury and Distress Scale (MIDS) [53] will be utilized to measure participant distress for having witnessed, done something, or failed to do something in the course of their work that bothers them. Reliability for the MIDS is excellent for healthcare workers (α = .95) and first responders (α = .94) [53].

#### Sleep quality.

This section will ask participants about their sleep quality using the Insomnia Severity Index (ISI) [54]. This is a seven-item self-report questionnaire used to determine the severity of insomnia symptoms in the past two weeks [54]. Each item is scored on a 5-point Likert scale, and the total score ranges from 0 to 28 [54]. The first three questions use a scale from 0 (none) to 4 (very severe) to rate particular sleep issues, such as trouble falling asleep, remaining asleep, and waking up too early [54]. The other four questions assess broader impressions of insomnia, such as sleep dissatisfaction, impairment, distress, and interference with everyday functioning, using labels ranging from “not at all” to “very much” [54]. Four levels of scores are distinguished: no clinically significant insomnia (0–7), subthreshold insomnia (8–14), moderate clinical insomnia (15–21), and severe clinical insomnia (22–28) [54].

#### Happiness.

We will use three items of the Subjective Happiness Scale [55]. This measure will allow participants to rate how happy they see themselves as a person and how happy they perceive themselves to be compared to their peers.

#### Loneliness.

We will use four items from the Revised UCLA Loneliness Scale [56], three items from the Existential Loneliness Scale [57], and eight items created for this study to measure cognitive/epistemic loneliness.

#### Resilience.

Resilience will be measured with a modified version of the Vicarious Resilience Scale [58] and the Social Support Index [34, adapted from 59], the brief version of the Emotional Self Awareness Scale [60], the Sense of Coherence scale adapted from Antonovsky (1993) and Schäfer et al., 2019 [61, adapted from 62].

The Vicarious Resilience Scale is a 27-item measure of positive impacts service providers may experience from their client’s resilience and recovery [58]. The scale measures 7 factors: 1) increased capacity for resourcefulness; 2) client-inspired hope; 3) increased consciousness about power and privilege relative to clients’ social location; 4) changes in life goals and perspective; 5) increased self-awareness and self-care practices; 6) increased recognition of clients’ spirituality as a therapeutic resource; and 7) increased capacity for remaining present while listening to trauma narratives. Each item is scored on a 6-point Likert scale ranging from 0 (did not experience this) to 5 (experienced this to a very great degree). Total scores range from 0–135, with higher scores representing greater vicarious resilience. The VRS has excellent internal consistency reliability (α = .94) for the scale overall and  .77 to  .86 within the subscales [58].

The VRS has been revised for this project. The VRS (Revised) has updated directions and revised wording on nine items to more easily apply to the subsamples included in this project.

The Social Support Index is a seven-item measure that allows participants to indicate to what extent they feel they can rely on community, friends, and family to help them when they are experiencing periods of stress [36, adapted from 58]. This index uses a 6-point Likert scale that ranges from 0 (strongly disagree) to 5 (strongly agree). Two items ask participants to indicate how many family members, and friends and/or coworkers that they can turn to for advice, support, or help if they need it.

The Brief Emotional Self-Awareness Scale is a 13-item self-report test that evaluates a person's ability to recognize and control their emotions as well as their level of self-efficacy in this area [61]. The measure highlights the importance of self-awareness in mental health and flexible coping mechanisms [61]. Participants are assessed on how often they experience or use different emotional self-regulation behaviours by rating each item on a 5-point Likert scale from 0 (None of the Time) to 4 (All of the Time) [59,61]. The questions evaluate critical components of emotional intelligence, such as emotional clarity, problem-solving skills, and impulsivity under pressure [61]. The questions also evaluate emotional difficulties, including trouble recognizing emotions, expressing sentiments, and having a propensity to overreact in emotionally sensitive circumstances [61].

The Sense of Coherence (SOC) Scale was designed to measure a participant’s confidence in their predictability in their external and internal environment and their ability to cope with negative, stressful experiences [60,62]. Across 16 studies that used the 13-item SOC scale, Cronbach’s alpha ranged from  .74 to  .91 [62]. Holmefur et al. (2014) [63] created a 7-item version of the SOC scale with a 7 point Likert scale response format ranging from 0 to 6. Two sample items include “Until now, your life has had” with a response continuum ranging from 0 = “No clear goals or purpose at all”, to 6 = “Very clear goals and purpose”, and “How often do you have the feeling that there’s little meaning in the things you do in your daily life?”, with 0 = “Very often” and 6 = “Rarely or never”. Holmefur et al. (2014) [63] reported a reliability coefficient of  .81.

#### Coping strategies.

This section will ask participants about the coping strategies or self-care they utilize by using the Mindful Self-Care Scale – Brief (MSCS-B) and Coping Styles [64].

The Mindful Self-Care Scale – Brief (MSCS-B) is a 24-item scale measuring the frequency of self-care behaviours. Participants will be asked to rate each item on a 5-point Likert scale based on the frequency they engaged with each behaviour in the past week, from 0 (never) to 5 (regularly). Items are categorized within the following six subscales: mindful relaxation, physical care, self-compassion and purpose, supportive relationships, supportive structure, and mindful awareness [65]. An average score is taken from each subscale to show the prevalence of self-care activities in different domains.

Coping Styles measures the prevalence of positive and negative coping strategies to stress. It contains 8 items presenting various coping responses from gratitude, to avoidance, to disengagement to the following statement, “when life becomes stressful, and I face difficult challenges…” [adapted from 66]. Participants will rate how accurate each statement is to them via a 5-point Likert scale, ranging from 0 (I don’t do this at all) to 4 (I do this a lot). Pilot testing of the 8-item Coping Styles measure found a Cronbach’s alpha of  .83 (Killian, [Unpublished]).

### Data analysis plan

This mixed-methods study will involve both quantitative and qualitative data. The size and diversity of the sample population will allow for multiple approaches to data analysis.

#### Quantitative analysis.

Quantitative data from the online survey will be uploaded to SPSS for statistical analysis. Scale items that require reverse scoring will be sorted appropriately once the dataset has been uploaded to SPSS. Descriptive statistics (frequencies, central tendencies, etc) will describe the sample and explore demographic and organizational variables. Inferential statistics will be used to identify relationships between variables.

#### Qualitative analysis.

Qualitative survey data and transcripts from interviews and focus groups will be uploaded to ATLAS.ti for coding. We will utilize thematic analysis to ensure the quality of analysis and transparency of the process. This methodology follows five steps: familiarizing yourself with the data, generating initial codes, searching for themes, reviewing themes, defining and naming themes, and producing the report [64]. Our qualitative analysis team will meet weekly to discuss new and emerging themes, which will help us identify when we have reached data saturation.

### Ethical considerations

Ethical approval for this study has been obtained through the institutional research ethics board of the first author’s institution. The primary ethical consideration for this project is the potential harm caused to participants when asked about their experience with exposure to trauma. To mitigate this potential harm, we will include free, accessible support resources at the start and conclusion of our online survey. All interviewers and focus group facilitators will have been trained in trauma-informed interviewing techniques. To protect the confidentiality and anonymity of research participants, we will not collect personal data from survey participants, and all identifying information will be removed from surveys at the point of data analysis. Identifying information will be removed from interviews and focus groups at the point of transcription.

We will also take measures to protect our research team from the impacts of conducting trauma research. Examples of such measures include team debriefing, incorporating self-care into the workday, and alternating tasks to allow for breaks from one area of focus.

### Timeline

We plan for active data collection to begin in the spring of 2026 and span eight months to allow for the maximum number of participants. Interviews will be scheduled throughout this timeframe as requests are made. Data analysis will take approximately four months after that, with knowledge dissemination beginning as soon as the analysis is completed.

## Discussion

### Strengths and limitations

Multiple modes of participation will be offered to participants, such as surveys, focus groups, and one-on-one interviews, which will allow for larger engagement with participants. This also allows flexibility in when participants wish to complete the aspects of the study, such as the survey portion, as many Public Safety Personnel do not work typical daytime shifts.

However, a potential limitation to this study could be the sensitive nature of the topics discussed throughout the survey and/or focus groups. To acknowledge this concern and keep our ethical considerations in mind, participants will be able to leave the survey at any point without penalty or pressure. An additional limitation that could be present is data saturation when we start the analysis portion of this study and find no new themes emerging from participants. This may provide the team reasons to believe the study has come to a logical conclusion.

### Amendments and withdraws

Participants will be informed ahead of the survey that they can leave questions blank and move to the subsequent question. Participants will be informed at the beginning and end of the survey that they can choose to withdraw at any point and not have their data used by contacting the researchers and making this request.

### Knowledge dissemination

Our team is committed to accessible knowledge dissemination. To that end, we have several knowledge dissemination activities planned that will allow us to share our findings with academics, organizations, and frontline personnel. Our knowledge dissemination plans include the following activities.

1. **Presentations:** We will present our findings at academic conferences, such as the Ontario First Responders’ Mental Health Conference and the Canadian Psychological Association and Canadian Sociological Association annual conferences.
2. **Training resources:** We will use our findings to create and share training resources for individuals and organizations.
3. **Publications:** We will submit articles to peer-reviewed academic journals.
